# The role of integrin family in bone metabolism and tumor bone metastasis

**DOI:** 10.1038/s41420-023-01417-x

**Published:** 2023-04-10

**Authors:** Liwei Mao, Lian Wang, Jiake Xu, Jun Zou

**Affiliations:** 1 School of Kinesiology, Shanghai University of Sport, 200438 Shanghai, China; 2 School of Biomedical Sciences, The University of Western Australia, WA 6009 Perth, Australia

**Keywords:** Mechanisms of disease, Metastasis

## Abstract

Integrins have been the research focus of cell-extracellular matrix adhesion (ECM) and cytokine receptor signal transduction. They are involved in the regulation of bone metabolism of bone precursor cells, mesenchymal stem cells (MSCs), osteoblasts (OBs), osteoclasts (OCs), and osteocytes. Recent studies expanded and updated the role of integrin in bone metabolism, and a large number of novel cytokines were found to activate bone metabolism pathways through interaction with integrin receptors. Integrins act as transducers that mediate the regulation of bone-related cells by mechanical stress, fluid shear stress (FSS), microgravity, hypergravity, extracellular pressure, and a variety of physical factors. Integrins mediate bone metastasis of breast, prostate, and lung cancer by promoting cancer cell adhesion, migration, and survival. Integrin-mediated targeted therapy showed promising prospects in bone metabolic diseases. This review emphasizes the latest research results of integrins in bone metabolism and bone metastasis and provides a vision for treatment strategies.

## Facts

- The integrin family is involved in the proliferation, differentiation, adhesion, and migration of BMSCs, OBs, OCs, and osteocytes.
- Integrins, as important transduction molecules, mediate a variety of biophysical stimuli to regulate bone metabolism.
- By mediating cell-ECM activity, integrins have become important target therapy strategies for bone metabolism-related diseases and the incubator for drug delivery system development.
- Integrin mediates bone metastasis of prostate cancer and breast cancer, promotes the development of osteosarcoma and lung metastasis, and is also a hotspot for the prevention and treatment of cancer progression.

## Open questions

- What is the molecular mechanism by which integrins regulate different intracellular signaling pathways?
- How do integrins receive and recognize mechanical and physical stimuli and transmit signals?
- How to promote cancer cell death and inhibit cancer cell metastasis by regulating integrins?

## Introduction

**Fig. 1:**
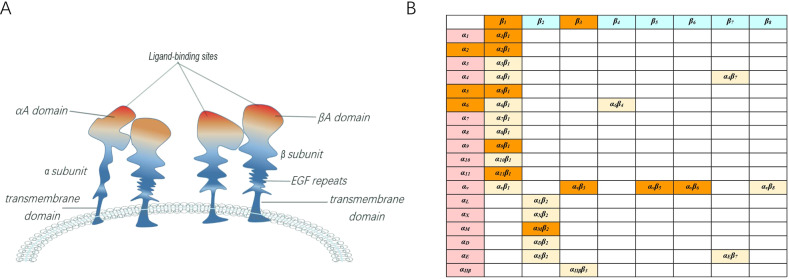
Schematic representation of the structure and subtypes of the integrin family. **A** Schematic of integrins infrastructure. **B** Integrin family subunits and 25 existing subtypes. Orange integrins are active in bone metabolism.

Integrins are stable noncovalent dimers found in mammals, consisting of 18 α and 8 β subunits independently. As an integrator, integrins activate downstream pathways, so-called ‘outside-in’ and ‘inside-out’ signaling, through ECM-cytoskeleton linkers formed after cell adhesion [1, 2]. Integrins are cell membrane protein receptors. α subunit consists of αA domain, β-propeller, thigh domain, calf-1 domain, calf-2 domain, transmembrane domain, cytoplasmic domain, and β subunit consists of βA domain, hybrid domain, plexin/semaphoring/integrin homology (PSI) domain, epidermal growth factor (EGF) repeats, β-tail domain, transmembrane domain and cytoplasmic domain. Different combinations of α and β subunits form 25 heterodimers with similar structures and distinct functions in mammals, of which β_1_ and α_V_ are the most common subunits constituting integrins (Fig. 1).

Integrins are bidirectional signal receiving and transmitting molecules. Integrins bind to intracellular inactivators in a bent, dull conformation. The balance between inactive and activated states determines the function of integrins [3]. Integrins are transformed into a high-affinity extended conformation of ECM when the intracellular signal-promoting protein and adaptor protein (talin and kindlin) are activated and bind to the cytoplasmic tail of the β-subunit [4]. Activated integrins form strong ligand binding with ECM and continuously recruit clusters. This process is termed “inside-out” signaling of integrin activation. After large amounts of integrins are recruited to the ECM, integrins trigger signals from outside to inside by recruiting large numbers of protein complexes containing proteases, protein scaffolders, and protein adapters [5, 6]. This process is termed “outside-in” signaling of integrin activation. Integrins can also enter cells through endocytosis to perform inside-out signaling by recruiting intracellular focal adhesion kinase (FAK) and returning to the cell surface again through exocytosis [7].

## Integrins regulate migration, adhesion, and differentiation of BMSCs

Mesenchymal stem cells (MSCs) are upstream progenitor cells with the ability to proliferate and differentiate [8]. Bone marrow mesenchymal stem cells (BMSCs) are important targets for studying bone-related diseases such as osteoporosis (OP), osteoarthritis (OA), and hyperostosis [9–12]. Integrins expression is dynamically regulated during BMSCs osteogenic differentiation. The expression of integrin α_2_ was significantly downregulated during osteogenic differentiation of hMSCs, while the expression of integrin α_3_ and α_V_ were up-regulated with the high expression of osteogenic markers [13]. These findings indicate that integrins can be used not only as biomarkers of osteogenic differentiation but also as essential regulators of bone metabolism.

Neural cell adhesion molecule (NCAM) can regulate the migration of BMSCs by activating cofilin through integrin β_1_ signaling to regulate the formation of directional lamellipodia at the initial stage of migration [14]. In vitro study showed that overexpression of integrin β_1_ promoted proliferation and survival of BMSCs in hypoxia microenvironment [15]. Upregulation of integrin β_1_ expression was also found during the treatment of low-intensity pulsed ultrasound (LIPUS) to promote fracture healing and chondrogenesis [16, 17]. An early study showed that integrin α_v_β_3_ was a key point for pre-osteoblasts and BMSCs precursors to break through matrix barriers and complete cell migration [18]. Bone sialoprotein (BSP) enhanced BMSCs migration by linking matrix metalloproteinase 2 (MMP-2) and integrin α_v_β_3_ to form complexes. Vitamin D was also confirmed to promote osteogenic differentiation of BMSCs by increasing the expression of integrin α_v_β_3_ [19]. Mitsuaki found that Wnt-induced secreted protein 1 (WISP-1) promoted BMSCs osteogenic differentiation by increasing the expression of bone morphogenetic protein 2 (BMP-2). Further study showed that WISP-1 as a ligand up-regulated the expression of integrin α_5_β_1_, and the deletion of integrin α_5_β_1_ significantly inhibited the osteogenic effect of WISP-1 [20]. Meanwhile, rat BMSCs transfected with integrin α_5_β_1_ enhanced cell adhesion, survival, migration, and NO production [21]. Integrin α_5_ expression was up-regulated during bone regeneration therapy, and simvastatin-induced osteogenic differentiation was significantly weakened after inhibition of integrin α_5_ expression [22]. Integrin α_2_β_1_ mediated the osteogenic effect of type II collagen (Col II) in BMSCs by activating Runt-related transcription factor 2 (RUNX2) through the integrin α_2_β_1_/FAK/c-Jun N-terminal kinase (JNK) signaling axis [23]. Decreased integrin α_2_ expression during aging is thought to affect BMSCs differentiation. Overexpression of integrin α_2_ increased RUNX2 and osterix levels and promoted osteogenic differentiation of BMSCs from elderly OP patients [24].

Recently, integrins were found to be involved in non-coding RNAs regulation of bone metabolism. Circular RNA VGLL3 (circRNA-VGLL3) promoted the osteogenic differentiation of adipose-derived mesenchymal stem cells (ADSCs) through circRNA-VGLl3/miR-326-5p/integrin α_5_ pathway [25]. Integrin α_5_ was also found to promote the survival and osteogenic differentiation of human periodontal ligament stem cells (hPDLSCs) as a target gene of miR-152-3p [26]. Integrins showed versatility in the early stage of bone metabolism by independently regulating migration and differentiation of BMSCs or mediating other pathways.

## Integrins regulate osteoblasts migration, differentiation, proliferation, and bone formation

Osteoblasts (OBs), derived from MSCs, are remodeling units of bone-forming cells and play an important role in the growth and maintenance of bone tissue [27]. The biological activities of OBs directly affect bone homeostasis, and integrins are active in multiple processes. Cellular communication network factor 1/2/3 (CCN1/2/3) promoted the formation of bone nodules in OBs culture. Integrin α_5_β_1_ and α_V_β_5_ were activated under the CCN3 stimulation [28]. CCN3-induced bone nodule formation and BMP-4 upregulation were inhibited by monoclonal antibodies to α_5_β_1_ and α_v_β_5_ integrin. Integrin α_v_β_1_ mediated the adhesion of OBs to CCN2 and promoted OBs maturation, bone nodule formation, and matrix mineralization [29]. CCN1 regulated parathyroid hormone receptor-1 (PTH1R) expression by interacting with α_V_β_3_ and/or α_V_β_5_ integrin complex, maintaining the homeostatic regulation of the PTH pathway during osteogenic differentiation [30].

As a receptor of type I collagen (Col I), integrin α_2_β_1_ deletion protected against age-related bone loss and biomechanical degeneration [31]. OBs culture with integrin α_2_β_1_ deletion revealed a significant elevation of Col I and osteogenic differentiation markers. However, integrin α_2_β_1_, as the primary receptor for lumican (a myogenic factor), was verified to play a significant role in promoting OBs differentiation through the extracellular signal-regulated kinase (ERK) pathway [32]. This demonstrates the functional diversity of integrins in the complex process of bone metabolism. Collagen XIII (Col XIII) is a kind of conserved transmembrane protein that regulates tissue metabolism and homeostasis [33, 34]. Integrin α_11_β_1_ recognizes two motifs of the Col XIII gene and mediates cell adhesion. The ligand-receptor complex played an apparent role in regulating bone metabolism homeostasis, and silencing of integrin α_11_β_1_ moderated the disruption of bone homeostasis caused by overexpression of Col XIII [35]. Furthermore, integrin α_11_β_1_ activated the Wnt pathway and promoted OBs differentiation by binding to osteolectin [36].

Increasing studies confirmed that β_1_ and β_3_ integrins are involved in various cytokine regulation processes during OBs differentiation. Integrin β_1_ regulated BMP-2-dependent signaling by positively regulating smad1/5 transcriptional activity during preosteogenesis [37]. Large conductance calcium-activated potassium channels were demonstrated to promote OBs differentiation and bone formation by binding to integrin β_1_ protein [38]. Epidermal growth factor-like repeats and discoidin I-like domain 3 (Edil3) was highly expressed in the process of OBs differentiation. It also promoted the expression of alkaline phosphatase, osteocalcin gene, RUNX2, and the phosphorylation of ERK. Inhibition of integrin α_5_β_1_ significantly attenuated Edil3-induced osteogenic differentiation [39]. In addition, fibronectin containing different external domains (A/B) was found to promote OBs differentiation and mineralization by binding to different integrins α_4_β_1_ and β_3_ [40, 41]. Vitronectin-derived peptide (VNP-16) regulated bone metabolism through OBs and osteoclasts by directly acting on different integrins [42]. VNP-16 directly interacted with integrin β_1_ and activated FAK to promote differentiation and viability of OBs. Meanwhile, VNP-16 inhibited the expression of OCs and preosteoclast maturation-related proteins by interfering with the integrin α_v_β_3_ signaling pathway. A subsequent study confirmed that integrins not only affected bone marrow but also regulated cortical bone development. Osterix activated OBs proliferation and promoted bone corticalization by enhancing integrin β_3_ transcription [43]. The development of bone cortex and femur length was impaired after silencing integrin β_3_. These findings confirm the complex and important role of integrins in OBs osteogenesis.

## Integrins regulate osteoclasts migration, differentiation, proliferation, and bone resorption

Osteoclasts (OCs) are another crucial modulator of bone metabolic homeostasis. On the one hand, osteoclast-mediated bone resorption is an important mechanism of bone loss diseases such as osteoporosis [44, 45]. On the other hand, the microenvironment formed by the OCs attachment site in bone tissue is the basis for OBs to exert bone formation. The large number of apoptotic bodies produced after OCs apoptosis is the end symbol of bone resorption and the beginning signal of osteogenesis [46–48]. The differentiation of OCs is mainly regulated by macrophage colony-stimulating factor (MCSF), receptor activator of NF-kappaB ligand (RANKL), and osteoprotegerin (OPG) [49–54]. For a long time, integrins have been found to be an important link in mediating OCs differentiation, proliferation, migration, and bone resorption.

The phenotype and number of OCs in ovariectomized mice were significantly affected by the deletion of integrin α_v_β_3_ [55]. Integrin α_v_β_3_ binds to the colony-stimulating factor-1 receptor (c-Fms) to form the cytoskeleton required for osteoclast migration. It activates the ERK/c-Fos signaling pathway to regulate cell adhesion, differentiation, and proliferation [56, 57]. Dual Ig domain-containing adhesion molecule (DICAM) preferentially binds to integrin β_3_ and inhibits the formation of integrin α_v_ and β_3_ dimers, thus impeding osteoclastogenesis in the downstream pathway [58]. The highly selective and competitive binding of integrin α_v_β_3_ with Arg-Gly-Asp (RGD) binding domain protein molecules can inhibit OCs differentiation and reduce bone resorption. Rhodostomin variants and Tablysin-15 are two effective integrins antagonists that inhibit ovariectomy (OVX) and LPS-induced osteoporosis without affecting the survival of other cells [59, 60]. Integrin α_v_β_3_-mediated actin rings are important structures that induce OCs migration and bone matrix adhesion. Phloretin and Tetraspanin 7 were found to inhibit OCs activity and reduced bone resorption by disrupting the actin cytoskeleton on the surface of OCs [61, 62]. Tatsuya et al. studied the regulatory effect of chondroitin sulfate-E (CS-E) on integrin α_v_β_3_ and its ligand. CS-E blocks the combination of the receptor-ligand complex by binding both integrin α_v_β_3_ and osteoactivin, which then inhibits OCs differentiation [63].

Integrin α_2_β_1_ acted on ameloblastin to promote OCs differentiation of bone marrow-derived monocytes (BMMCs) by enhancing cell adhesion and actin ring formation. Blocking integrin α_2_β_1_ moderated the osteoclastogenesis effect of ameloblastin and inhibited bone resorption [64]. Th17 cells are key effectors of inflammation and tissue damage, expressing both IL-7R and integrin α_2_β_1_ [65–69]. IL-7 enhanced the adhesion of Th17 cells to collagen through integrin α_2_β_1_, promoting IL-17 production and OCs function. Blocking integrin α_2_β_1_ inhibited IL-7-induced OCs differentiation and inflammatory bone resorption by reducing Th17 cell count and IL-17 production [70]. Similarly, cooperation between IL-7R and integrin α_1_β_1_ drives T cells-mediated bone loss by up-regulating the production of RANKL [71]. In addition, integrin α_9_β_1_ was confirmed to promote bone resorption. Gene deletion of integrin α_9_β_1_ increased trabecular bone and total bone volume in mice [72]. Integrin α_M_β_2_ was illustrated to promote osteoclastogenesis by enhancing the bone adhesion ability of classical monocytes [73]. These studies showed positive effects of integrins in maintaining OCs differentiation and proliferation.

Conversely, some integrin subtypes were inhibitors of OCs differentiation. An animal study showed that integrin α_v_β_5_ gene deletion significantly increased the number of OCs in bone tissue, both wild-type and OVX mice [74]. Exogenous supplementation of irisin (skeletal muscle-secreted myokine) was verified to promote osteoclastogenesis, and the enhancement effect of irisin was inhibited by integrin α_v_β_5_ neutralizing antibody [75] (Table 1).

**Table 1:** The role of integrins in bone metabolism at different cellular stages.

Integrins	Cell types	Potential pathways or binding protein	Regulating effects	Ref.
α_v_β_3_	BMSCs	BSP/MMP-2/α_v_β_3_	Promote cell migration	[18]
β_1_		NCAM/β_1_/cofilin	Regulate migration and promote proliferation	[14]
α_5_β_1_		WISP-1/α_5_β_1_/BMP-2	Enhance cell adhesion, survival and migration	[20]
α_2_β_1_		Col II/α_2_β_1_/FAK-JNK	Increase bone formation and defect healing	[23]
α_5_	ADSCs	circRNA-VGLl3/microRNA-326-5p/α_5_	Promote osteogenic differentiation	[25]
	hPDLSCs	microRNA-152-3p/α_5_	Promote survival and osteogenic differentiation	[26]
α_5_β_1_, α_V_β_5_	osteoblast	CCN3/α_5_β_1_, α_V_β_5_/BMP-4	Promote cell maturation, adhesion and matrix mineralization	[28]
α_v_β_1_		α_v_β_1_/CCN2/FAK/ERK	Enhance the adhesion and differentiation of osteoblasts	[29]
α_V_β_3_, α_V_β_5_		CCN1/α_V_β_3_, α_V_β_5_/ PTH1R	Promotes bone anabolism through activation of PTH signaling	[30]
α_2_β_1_		Col I/α_2_β_1_, α_2_β_1_/ERK	Regulate cell differentiation	[31, 32]
α_11_β_1_		Col XIII/α_11_β_1_, α_11_β_1_/osteolectin/Wnt	Promote cell adhesion and differentiation	[35, 36]
β_1_		β_1_/Smad/BMP2	Regulate osteogenic differentiation	[37]
α_5_β_1_		EDIil3/α_5_β_1_/ERK	Up-regulate osteogenic factors	[39]
α_4_β_1_, β_3_		Fibronectin/α_4_β_1_, β_3_	Promote cell mineralization and differentiation	[40, 41]
β_1_, α_V_β_3_		VNP-16/β1/ FAK, VNP-16/α_V_β_3_	Promote osteoblast differentiation and survival. Inhibit the expression of osteoclast maturation-related proteins.	[42]
α_V_β_3_	osteoclast	α_V_β_3_/c-Fms/ERK/c-Fos	Regulate cell adhesion, differentiation and proliferation	[56, 57]
		DICAM/α_V_β_3_	Inhibit osteoclastogenesis	[58]
		Phloretin, Tetraspanin 7/α_V_β_3_	Inhibite osteoclasts activity by disrupting the actin cytoskeleton	[61, 62]
		CS-E/α_V_β_3_, osteoactivin	Inhibit osteoclast differentiation	[63]
α_M_β_2_			promote osteoclastogenesis	[73]
α_2_β_1_		α_2_β_1_/Ameloblastin	Inhibit osteoclast differentiation	[64]
		α_2_β_1_/ IL-7/IL-17	Inhibit osteoclast differentiation and proliferation	[70]
α_1_β_1_		α_1_β_1_/ IL-7R	Drive T cells-mediated bone loss	[71]
α_9_β_1_			Promote bone resorption	[72]
α_v_β_5_		α_v_β_5_/Irisin	Inhibit osteoclastogenesis	[74, 75]

## Integrins mediate mechanotransduction and regulate bone metabolism

Exercise promotes bone formation and prevents various pathologic bone loss [76, 77]. Lack of physical activity and exercise has become an important cause of bone loss disease [78, 79]. Cellular molecular studies have partly revealed the mechanism by which exercise promotes bone formation. Mechanical and physical stimuli, such as pressure, tension, fluid shear stress (FSS), ultrasound, and electrical stimulation, increase bone formation by promoting osteogenic differentiation and proliferation of BMSCs, OBs, and osteocytes [80–85]. The microenvironment of microgravity created by the rotator inhibited osteogenic differentiation and bone mineral formation [86, 87]. These studies demonstrated the sensitivity of bone-associated cells in sensing, receiving, conducting, and transforming mechanical stimuli into intracellular signals.

**Fig. 2:**
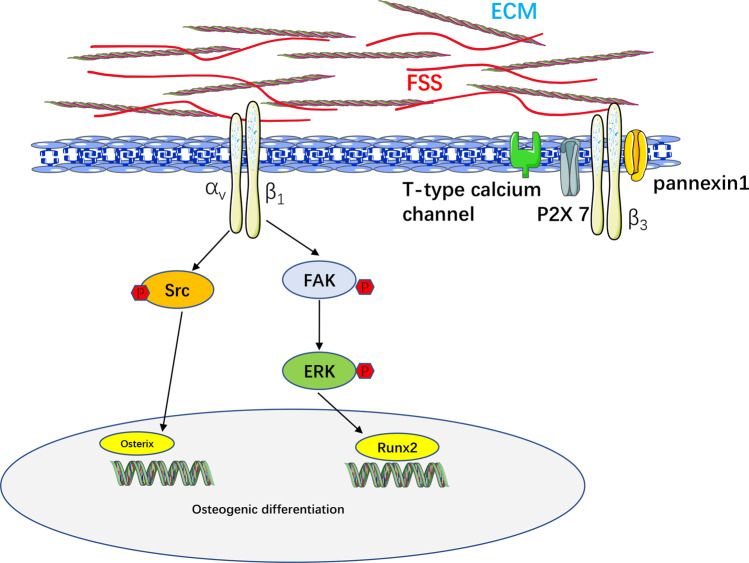
Integrins mediate FSS to regulate bone metabolism. FSS activates integrin α_v_β_1_ and promotes Src, FAK, and ERK phosphorylation to enhance osteogenic differentiation. After FSS stimulation, a large number of ion channels are located around β_3_ integrin in the osteocyte membrane, forming a potential mechanical conduction complex.

**Fig. 3:**
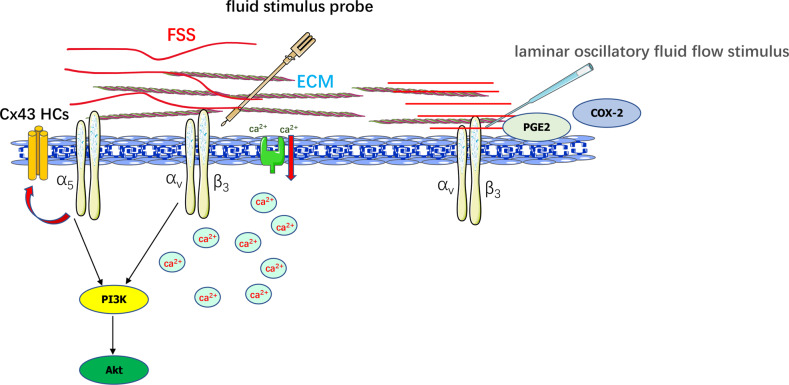
Exogenous FSS stimulates integrins to regulate osteocyte activity. Integrin α_V_β_3_ promotes Ca^2+^ signaling pathway diffusion, activates PI3K/Akt signal pathway, and increases the generation of anabolic factors. FSS stimulates α_5_ integrin and activates Cx43 HC.

Researchers identified different types of mechanosensitive channels on the surface of bone cells, including classic Piezo and transient receptor potential vanilloid (TRPV) channels [88–91]. Osteocytes, OBs, and MSCs in the lacunar-canalicular system are the main cells for the transduction of mechanical and physical stimuli. The morphological structure and volume of mouse cranial OBs changed significantly after pulsed fluid flow and the RNA and protein expression of integrin α_5_ increased [92]. FSS simulated by the perfusion system significantly increased the expression of osteogenic markers, as well as the phosphorylation levels of ERK1/2, RUNX2, and FAK in hMSCs. FSS promoted the expression of β_1_ integrin, and the osteogenic effect of FSS was inhibited by blocking integrin β_1_ [93]. Targeted deletion of integrin α_V_ leads to reduced cell reaction to FSS and impaired Src phosphorylation [94]. Superresolution microscopy showed that the membrane proteins implicated in mechanical conduction were preferentially located near integrin β_3_ after FSS stimulation in osteocytes [95]. In addition, multiple mechanotransduction ion channels, including pannexin1 channel, purinergic receptor P2X 7, and T-type calcium channel, were located in the vicinity of integrin β_3_, forming a potentially specific mechanical conduction complex (Fig. 2). Osteocytes stimulated with fluid stimulus probe showed accelerated ca^2+^ expansion, and ca^2+^ signaling pathway diffusion was inhibited by EMC ATP scavenger and integrin α_v_β_3_ blocker [96]. Matthew found that laminar oscillatory fluid flow stimulus on osteocytes can promote anabolism, and blocking integrin α_v_β_3_ resulted in osteocytes morphology destruction, reduced expansion area, process retraction, and decreased anabolic factors release [97]. Connexin43 hemichannels (Cx43 HCs) are important mechanical sensing channels that regulate the release of bone anabolic molecules by osteocytes [98]. FSS stimulated Cx43 HCs to open and release anabolic factors by activating osteocyte α_V_ and α_5_ integrins. Further study showed that blocking integrin α_V_ inhibited the PI3K/Akt signaling pathway, which in turn inhibited the activation of integrin α_5_ and Cx43 HCs opening [99] (Fig. 3).

**Fig. 4:**
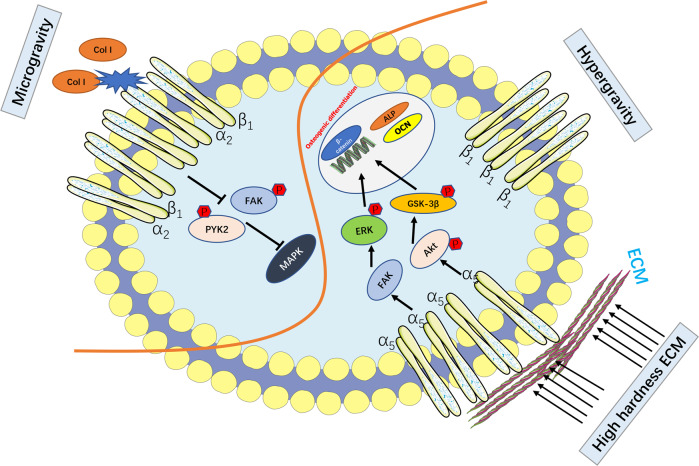
Integrins mediate changes in the cellular microenvironment to regulate bone metabolism. Microgravity inhibits the binding of Col I to integrin α_2_β_1_ and inhibits the phosphorylation of FAK and PYK2. Hypergravity promotes integrin β_1_ gathering on the surface of osteoblasts. ECM stress (hardness) activates integrin α_5_ and promotes the phosphorylation of key osteogenic factors.

Microgravity stimulation inhibited osteogenic differentiation of hBMSCs by decreasing Col I expression and damaging interactions of Col I and integrin α_2_β_1_ [100]. Conversely, hypergravity stimulation increased the concentration of integrin β_1_ on the membrane of osteoblastic cells [101]. It is worth noting that the overall expression levels of integrin β_1_ did not change in response to hypergravity stimulation, and this may be an active gathering of integrins after sensing stimulus. Interestingly, both hypergravity and microgravity induced integrin β_1_ enrichment, but the opposite effects deserve further investigation. The hardness of ECM affected hBMSCs osteogenic differentiation under pressure 62–68 kPa. The expression of integrin α_5_ and bone anabolic factors (col1α1, RUNX2, osteocalcin) increased with the improvement of ECM hardness, suggesting that matrix stiffness affected the osteogenesis of hMSCs through integrin α_5_-mediated mechanical transduction [102] (Fig. 4).

**Fig. 5:**
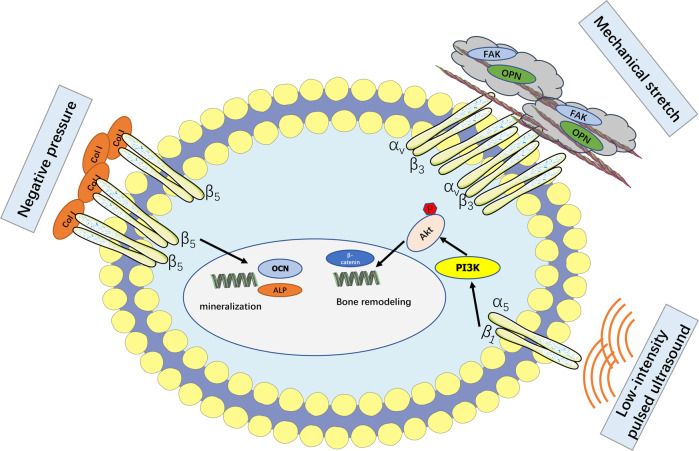
Integrins mediate physical factors to regulate bone metabolism. Negative pressure therapy enhances the binding of Col I and β_5_ integrin to promote cell mineralization. Mechanical stretch promotes the plaque mineralization of integrin α_V_β_3_-matrix interaction sites. Low-intensity ultrasound enhances bone remodeling by activating integrin α_5_β_1_ and promoting Akt phosphorylation.

Physical factor therapy is also an effective method for bone regeneration [103]. Early studies found discrepant electrophysiological responses of human bone cell membranes to different frequencies of mechanical stress. Depolarization and hyperpolarization after mechanical stimulation were inhibited by integrin α_v_, α_5_, β_1_, and β_5_ blockers [104]. Negative pressure wound therapy (NPWT) has been recognized as an effective method for healing bone injury [105–107]. Cell experiments demonstrated that NPWT promoted the proliferation and osteogenic differentiation of periosteum-derived MSCs. The expression of integrin β_5_, Col I, and osteocalcin increased during the process, along with increased alkaline phosphatase activity and cell mineralization [108]. As a type of phototherapy, 635 nm LED irradiation significantly inhibited the maturation of mouse OCs by reducing integrin β_3_ expression and disrupting actin structure [109]. Low-intensity pulsed ultrasound stimulation (LIPUS) promoted OBs proliferation, differentiation, and bone formation by activating β-catenin, P-Akt, Bcl-2, and downstream pathways. Blocking integrin α_5_β_1_ inhibited the LIPUS-induced osteodifferentiation [110]. Mechanical stretching activated integrin α_v_β_3_ and increased the number and size of plaques at integrin adhesion sites in osteoblasts [111]. These studies suggested the potential role of integrins in promoting bone regeneration by mediating physical factor therapy (Fig. 5).

## Integrins are participants and therapeutic targets in bone metastases

### Breast cancer

Organotropic metastases have always been the main obstacle to conquer in cancer treatment. Proteomics revealed the organ-selected specificity of tumor exosome-derived integrins, in which exosome integrin α_6_β_4_ and α_6_β_1_ were associated with lung metastasis, while integrin α_v_β_5_ was associated with liver metastasis [112]. Breast cancer is highly likely to cause osteolytic disease by releasing OCs growth factor into bone microcirculation [113, 114]. In fact, bone metastasis is the main cause of death and morbidity of breast cancer, accounting for more than 70% of metastasis, and specific integrins play an important role [115].

Integrin β_3_ was verified to be an important factor in early bone and soft tissue metastasis of breast cancer, and its inhibitors are recommended for early intervention [116]. Cancer cells with high expression of integrin β_3_ exhibited metabolic abnormalities, including enhanced oxygen consumption, reactive oxygen species, and protein production [117]. mTORC1 is a key target of integrin β_3_-mediated metabolic abnormalities. The level of integrin β_3_ in peripheral blood exosomes and vesicle-incubated cells increased in the breast cancer mouse model. Cell proliferation and migration decreased significantly, and osteolytic lesions were reversed after conditional deletion of integrin β_3_ [118]. Based on this, researchers developed a micellar nanoparticle that specifically recognizes integrin β_3_ and is loaded with chemotherapy drugs for targeted therapy [119].

Integrin α_2_β_1_ showed different biological effects in different stages of tumorigenesis and metastasis. In vivo experiment of bone metastasis in breast cancer showed that overexpression of integrin α_2_β_1_ promoted the growth and spread of tumor in situ but did not increase bone destruction, whereas decreased expression of integrin α_2_β_1_ increased osteolysis in bone tumors [120]. This provides an important basis for the staged treatment of breast cancer. Integrin α_5_ was found to exacerbate bone metastasis by promoting cancer cell adhesion, migration, and survival [121]. Besides, integrin α_5_ mediated RUNX2 to promote bone attraction and adhesion of breast cancer cells [122]. High expression of integrin α_5_ was detected in bone metastases from renal cell carcinoma, with increased Akt and FAK activity and decreased PTEN expression [123]. Integrin α_v_, β_1_, and β-like 1 are key contributors to bone metastasis of breast cancer. By mediating TGF-β signaling, integrins promoted the recruitment, retention, and growth of oncocytes in the bone microenvironment, and the development of integrin inhibitors has become an important means of tumor treatment and bone metastasis prevention [124–126].

In recent years, many new mechanisms of integrins in the process of bone metastasis in breast cancer have been discovered. At the transcriptional level, enhancer of zeste homolog 2 (EZH2) up-regulated integrin β_1_ transcription and further activated FAK. FAK enhanced TGF-β receptor phosphorylation, thereby activating the TGF-β pathway and promoting bone metastasis in breast cancer [127]. Integrins α_5_ and β_3_ were found to be target genes of the miRNA-30 family. miRNA-30 effectively weakened the invasion of breast cancer cells to bone tissue by directly inhibiting integrin α_5_ and β_3_ [128]. As receptors, integrin α_v_β_3_ interacted with BSP to promote bone metastasis of breast cancer, which is an important link in regulating the bone metastasis cascade of breast cancer [129]. In addition, integrin α_4_β_1_ was found to bind to the cognate ligand vascular cell adhesion molecule 1 (VCAM-1) to promote the recruitment of monocyte osteoclast progenitors and enhance local osteoclast activity [130]. Intercellular adhesion molecule 1 (ICAM1) is an important regulator of tumorigenesis and metastasis. Multiple integrin receptors (integrin α_2_, α_L_, α_M_, α_V_, β_2_, β_6_) were shown to mediate the process by which ICAM1 promotes bone metastasis in breast cancer through TGF-β/SMAD/epithelial-to-mesenchymal transition signaling [131].

### Prostate cancer

Bone metastasis, as an important cause of death in prostate cancer, is also a great challenge in tumor treatment [132]. Cancer cells enter the bone microenvironment and change the original bone structure and function through a multi-step process including colonization, dormancy, regeneration and development, and reconstruction [133, 134]. Integrins are involved in several stages of bone metastasis in addition to dormancy.

Integrin β_1_ was significantly activated during bone metastasis of prostate cancer and increased metastasis to lymph nodes and bone [135, 136]. Homeobox B13 (HOXB13), a transcription factor of prostate cancer cells, regulated the long noncoding RNA HOXA11-AS to promote the transcription level of integrin α_V_β_1_ and aggravate bone metastasis [137]. In addition, methyltransferase-like 3 (METTL3), which is highly expressed in prostate cancer cells, up-regulated integrin β_1_ transcription under the action of m6A-RNA binding protein human antigen R. The high affinity of integrin β_1_ and Col I promoted bone metastasis [138]. Bone metastases from prostate cancer have a specific affinity for bone Col I, which distinguishes them from other visceral metastases. This affinity attachment was regulated by integrin α_2_β_1_, and integrin α_2_β_1_ antibodies inhibited cell binding to Col I [139, 140]. Phosphorylated adaptor protein Talin1 enhanced bone metastases of cancer cells by activating integrin β_1_ [141]. Tenascin-C is an important component of OBs ECM, which promotes the colonization and development of trabecula metastases [142]. The affinity of integrin α_9_β_1_ for Tenascin-C enables selective migration and colonization of carrier cells to Tenascin-C-rich bone tissue. Blocking affinity proteins or integrins has a positive effect on the prognosis of prostate cancer patients with bone metastases. Melatonin MT1 receptor effectively inhibited the expression of integrin α_2_β_1_ and the transcriptional activities of FAK, C-SRC, and NF-κB, thereby reducing the migration and invasion ability of prostate cancer cells [143]. In addition, interference with integrin β_1_ was shown to reduce bone metastasis in prostate cancer and improve the prognosis of cancer radiotherapy [144].

As mentioned above, integrin α_v_β_3_ is an important regulatory molecule mediating OCs activity. Activation of integrin α_v_β_3_ on prostate cancer cells is critical for the recognition of key bone-specific matrix proteins [145]. In metastatic prostate cancer cells, integrin α_v_β_3_ supported osteoclastogenesis through RUNX2/Smad5 phosphorylation and NF-κB ligand signaling activation [146]. Integrin α_v_β_3_ has thus become a center for targeted drug delivery or therapy for bone metastases in prostate cancer. By constructing integrin α_v_β_3_ ligands and using a liposome drug delivery system, osteolytic lesions caused by bone metastasis can be effectively alleviated [147]. The delivery system also significantly reduced cancer pain and prolonged survival in mice. D-pinitol was also confirmed to reduce the migration and invasion of cancer cells by inhibiting the expression of integrin α_v_β_3_ on the surface of prostate cancer cells [148].

In prostate cancer cells, highly expressed prostate stem cell antigen (PSCA) interacted with growth factor progranulin (PGRN) to up-regulate integrin α_4_ transcription and activate the NF-κB pathway. The NF-κB/integrin-α_4_ pathway promoted the adhesion of prostate cancer cells to bone marrow endothelial cells (BMECs) [149]. WISP-1 enhanced the expression of VCAM-1 in prostate cancer cells and promotes the expression of integrin α_4_β_1_ in osteoblasts via MAPK pathway [150]. WISP-1/VCAM-1/integrin α_4_β_1_ axe promoted the adhesion of prostate cancer cells to osteoblasts. Blocking α_6_ integrin significantly reduced the progression of prostate tumor bone metastasis and inhibited osteolytic lesions [151]. At the same time, integrin α_v_β_6_ was found to be involved in the osteolysis process secondary to prostate tumors by selectively inducing metalloproteinase 2 (MMP2) to increase bone matrix degradation [152].

### Lung cancer

Bone tissue is one of the most common target sites of distant metastasis of lung cancer. The incidence of bone metastasis in lung cancer is 30–40%, and the average survival time after metastasis is 6 to 10 months [153]. It was found that integrin β_3_ expression was increased in SBC-5 cells (a specific bone-metastatic small cell lung cancer cell). Inhibition of integrin β3 downregulated the adhesion, migration, and invasion of cancer cells [154]. Moreover, integrin α_v_β_3_ was shown to mediate bone metastases in lung cancer by binding ligand periostin [155]. Silencing integrin α_v_β_3_ inhibited periostin-mediated cancer cell proliferation, migration, and invasion. The number of osteoclasts, bone damage, and Ca^2+^ concentration was significantly reduced in the bone metastasis model.

### Osteosarcoma

Given the important role of integrins in bone metabolism, integrins were also active in both in situ and metastatic osteosarcomas. Integrin β_1_ was up-regulated in metastatic osteosarcoma tissues and activated the NF-κB signaling pathway [156]. High expression of integrin β_1_ was associated with poor prognosis, and inhibition of integrin β_1_ increased apoptosis of osteosarcoma cells. Anti-β_1_ integrin monoclonal antibody AIIB2 significantly inhibited pulmonary metastasis of osteosarcoma cells but did not inhibit primary tumor growth [157]. Similar to bone metastasis of prostate cancer, Tenascin-C and its receptor integrin α_9_β_1_ were essential factors for lung metastasis of osteosarcoma cells by mediating transcription gene YAP [158]. In addition, blocking integrin α_2_β_1_ reduced Col I binding and directly inhibited the proliferation and tumorigenic ability of primary osteosarcoma cells through JAK/STAT3 signaling [159].

**Fig. 6:**
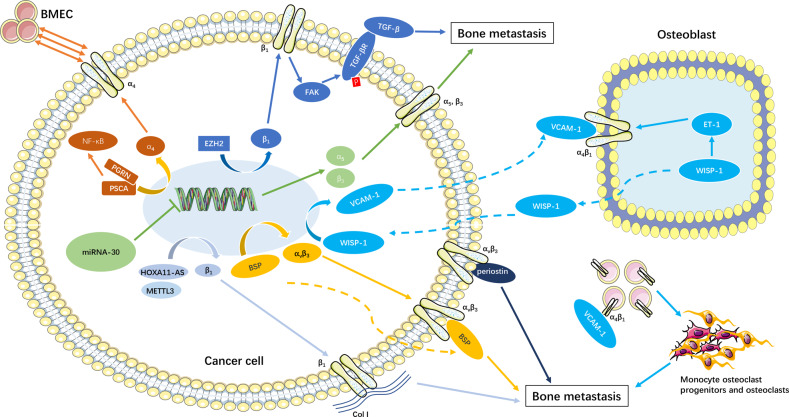
Schematic diagram of integrins regulating bone metastasis of cancer cells. High expression of Lnc HOXA11-AS, METTL3, and EZH2 in cancer cells increased the transcription level of integrin β_1_. Integrin β_1_ promoted bone metastasis through its high affinity with type I collagen and the FAK/TGF-β pathway. PSCA interaction with PGRG increased integrin α_4_ transcription levels and activated NF-κB, which promoted cancer cell affinity with BMECs. The miRNA-30 family inhibited integrin α_5_ and β_3_ transcription, The miRNA-30 family inhibited integrin α5 and β3 transcription, thereby attenuating bone metastasis of cancer cells. High expression of BSP in cancer cells increased integrin α_v_β_3_ expression and promoted bone metastasis by binding to integrin α_v_β_3_ as a ligand. Osteoblast-derived WISP-1 increased integrin α_4_β_1_ expression and induced cancer cells to overexpress VCAM-1. VCAM-1 binding with integrin α_4_β_1_ enhanced the adhesion of cancer cells to bone tissue. In addition, VCAM-1 activated in the micrometastasis microenvironment adhered to integrin α_4_β_1_-positive osteoclast progenitors, leading to local recruitment of osteoclast progenitors and osteoclasts in the bone, which exacerbates osteolysis.

Recently, studies confirmed that integrins were involved in the disease progression of osteosarcoma as target genes of non-coding RNAs. TargetScan prediction and dual luciferase reporter assay confirmed the target relationship between miR-127-3p and integrin α_6_. miR-127-3p inhibited osteosarcoma cell proliferation, invasion, migration, and survival by restraining integrin α_6_ [160]. Long non-coding RNA SNHG16 and integrin α_6_ were significantly up-regulated in osteosarcoma, while miR-488 was decreased. SNHG16 released integrin α_6_ expression through competitive sponge adsorption of miR-488 to promote osteosarcoma cell migration, invasion, and epithelial-mesenchymal transition [161] (Table 2, Fig. 6).

**Table 2:** The role of integrins in bone metastases of different cancers.

Cancer	Integrins	Role in tumor and bone metastasis	Ref.
Breast cancer	β_3_	Mediate the proliferation and migration of tumor cells and promotes osteolytic lesions	[117, 118]
	α_2_β_1_	Promote the growth and diffusion of tumor in situ and lytic lesion of bone tumor	[120]
	α_5_	Promote bone metastasis by promoting tumor cell adhesion, migration, and survival	[121–123]
	α_v_, β_1_	Promote the recruitment, retention and growth of oncocytes in the bone microenvironment	[124–126]
Prostate cancer	α_2_β_1_	Mediate Col I-dependent bone metastasis	[139, 140]
	β_1_	Mediate Talins phosphorylation to promote bone metastases	[141]
	α_9_β_1_	Affinitive bonding to Tenascin-C and promote selective migration and colonization	[142]
	α_v_β_3_	Identify key bone-specific matrix proteins that promote osteoclastogenesis and lytic lesions	[145, 146]
	α_4_, α_6_	Enhanced the adhesion ability and osteolytic lesions	[149, 151]
	α_v_β_6_	Mediate MMP2 to promote bone matrix degradation	[152]
Lung cancer	β_3_	Promote bone metastasis by promoting tumor cell adhesion, migration, and invasion	[154]
Osteosarcoma	β_1_	Promote apoptosis and inhibit lung metastasis by blocking β_1_ integrin expression	[157]
	α_9_β_1_	Mediate Tenascin-C to promote lung metastasis	[158]
	α_2_β_1_	Mediate Col I-dependent proliferation and tumorigenic ability	[159]
	α_6_	Promote cancer cell migration and invasion as non-coding RNA target gene	[160, 161]

## Conclusion and prospect

From its discovery to recent years, people have gradually deepened their understanding of the integrin family. As receptors for many ECM proteins, integrins are extremely active in the physiological and pathological studies of bone metabolism by mediating cell-ECM interactions. Integrins are involved in almost all cell life activities of MSCs, OBs, and OCs, while different subtypes of integrins have distinct biological effects based on diverse bone microenvironments. As a mechanosensing molecule, integrins promote bone formation by mediating different mechanical and physical stimuli. The active effects of integrins in breast cancer, prostate cancer, lung cancer and osteosarcoma are important therapeutic targets and have brought numerous clinical benefits. As a guide and modulator for cell-ECM interactions, integrins showed promise in the development of drug carrier systems and targeted delivery systems.

In the future, integrins have great potential for further research and utilization, not limited to bone metabolic diseases. The role of integrins in different subtypes needs to be further explored, and the interaction between integrins is also worth exploring. Mechanical transduction-induced osteogenesis mediated by integrins opens up a new research direction of exercise promoting bone metabolism health. The development of new delivery systems based on the targeting effect of integrins is a crucial approach for the treatment of many diseases including but not limited to bone metabolic diseases and cancers. There is still a big gap between existing research findings and clinical applications.

## Data Availability

All data included in this study are available upon request by contact with the corresponding author.
